# Bone and Cartilage Interfaces With Orthopedic Implants: A Literature Review

**DOI:** 10.3389/fsurg.2020.601244

**Published:** 2020-12-21

**Authors:** Remigiusz M. Grzeskowiak, Jim Schumacher, Madhu S. Dhar, David P. Harper, Pierre-Yves Mulon, David E. Anderson

**Affiliations:** ^1^Large Animal Clinical Sciences, University of Tennessee College of Veterinary Medicine, Knoxville, TN, United States; ^2^The Center for Renewable Carbon, Institute of Agriculture, University of Tennessee, Knoxville, TN, United States

**Keywords:** implant, bone, cartilage, cartilage scaffold, orthopedic implant, osseointegration, implant and tissue interface, biomechanics

## Abstract

The interface between a surgical implant and tissue consists of a complex and dynamic environment characterized by mechanical and biological interactions between the implant and surrounding tissue. The implantation process leads to injury which needs to heal over time and the rapidity of this process as well as the property of restored tissue impact directly the strength of the interface. Bleeding is the first and most relevant step of the healing process because blood provides growth factors and cellular material necessary for tissue repair. Integration of the implants placed in poorly vascularized tissue such as articular cartilage is, therefore, more challenging than compared with the implants placed in well-vascularized tissues such as bone. Bleeding is followed by the establishment of a provisional matrix that is gradually transformed into the native tissue. The ultimate goal of implantation is to obtain a complete integration between the implant and tissue resulting in long-term stability. The stability of the implant has been defined as primary (mechanical) and secondary (biological integration) stability. Successful integration of an implant within the tissue depends on both stabilities and is vital for short- and long-term surgical outcomes. Advances in research aim to improve implant integration resulting in enhanced implant and tissue interface. Numerous methods have been employed to improve the process of modifying both stability types. This review provides a comprehensive discussion of current knowledge regarding implant-tissue interfaces within bone and cartilage as well as novel approaches to strengthen the implant-tissue interface. Furthermore, it gives an insight into the current state-of-art biomechanical testing of the stability of the implants. Current knowledge reveals that the design of the implants closely mimicking the native structure is more likely to become well integrated. The literature provides however several other techniques such as coating with a bioactive compound that will stimulate the integration and successful outcome for the patient.

## Introduction

The implant-tissue interface refers to a transition zone between the surface of a surgical implant and adjacent tissue. It is a complex and dynamic environment characterized by mechanical and biological interactions between the implant and surrounding tissue which contribute to the quality of the interface. The ultimate goal for the implant is to become fully integrated with the tissue resulting in the long-term stability of the implant-tissue interface. The implant-tissue interfaces have been widely studied in various tissues. Numerous factors of importance contribute to this process and affect implant stability. The stability of the implant can be defined as primary (mechanical) and secondary (biological integration) stability. The primary stability is achieved by the implant immediately after its placement within the tissue and is greatly affected by the conditions during the implantation process. Secondary stability is obtained by the implant over time through the integration process. Successful integration of an implant within the tissue depends on both stabilities and is vital for short- and long-term surgical outcomes.

Integration of an implant within the tissue relies not only on the implant itself but also on the quality of the surrounding tissue. The process of inserting implant results in tissue injury causing bleeding into the implantation site and stimulation of the wound healing process. Bleeding is the first and most relevant step of the healing process because blood provides growth factors and cellular material necessary for tissue repair. The blood cells and platelets form a fibrin matrix around the implant, which serves as a scaffold for the regenerating tissue. As the tissue heals, the implant becomes integrated, strengthening the implant-tissue interface. Well-vascularized tissues heal more efficiently, and the progression of implant integration is more efficient as compared with avascular tissues such as cartilage where the implantation process is more challenging.

Advances in research aim to improve implant integration resulting in enhanced implant and tissue interface. Several methods have been employed to improve the process. This review provides a comprehensive discussion of current knowledge regarding implant-tissue interfaces within bone and cartilage as well as novel approaches to strengthen the implant-tissue interface.

## Orthopedic Implant and Bone Interface

The integration of an osseous tissue implant relies on the implant's capacity for osteoinduction, osteoconduction, and osseointegration (1–5). Osteoinduction is the ability of the implant to stimulate undifferentiated and pluripotent cells to transform into osteogenic cell lines (2). Osteoconduction is the implant's ability to allow cells to attach, proliferate and migrate along the implant surface and support deposition of bone on its outer surface and, where appropriate within pores and interconnected channels of the implant (2–5). Osteoinduction and osteoconduction occur simultaneously, under *in-vivo* conditions because healing of tissue injury during placement of the implant triggers the extracellular bone healing cascade which causes the release of osteoinductive growth and differentiation factors (2, 6, 7).

Osseointegration is important for healing at the interface between the bone and the implant (1–5), and its importance in the incorporation of implants used in orthopedics and orthodontics has been widely studied (5, 8–13). In the 1950s, the concept of osseointegration of implants was described by Brånemark et al. and Albrektsson et al. (2). The osseointegration of implants was evaluated using light microscopy and it was defined as direct contact between bone and implant (14, 15). Direct bone formation on the implant surface must be within a range between 10 and 20 μm for an implant to be considered well osseointegrated (2). Osseointegration occurring directly on the implant is termed direct osteogenesis and that occurring from surrounding bone is termed distance osteogenesis (1). The extent of osseointegration is associated with the implant's osteoinductive and osteoconductive characteristics (1, 2). The biomechanical definition of osteointegration describes osseointegration as a process leading to rigid fixation of an implanted alloplastic material during physiologic loading of the tissue into which it has been implanted (16). Recently, osseointegration has been further defined as a foreign body reaction where the interfacial bone is formed as a defense reaction to isolate the implant from the surrounding tissues (17). Failure of osseointegration of an implant leads to aseptic loosening of the implant.

In a recent study, investigators evaluated the postoperative follow-up of patients with cementless titanium implants used in total hip arthroplasty (THA). Implant survival rate was reported to be 85% at 10 years and 70% at 15 years after implantation (18, 19). Another study reported the survival rate of the implants used for total knee arthroplasty (TKA) of 90% 10 years after the surgery (20). Arthroplasty revision surgeries commonly have been required as a result of aseptic loosening of the implants, accounting for nearly 55% of THA revisions, and 35% of TKA revisions (19, 21). A prospective study reporting the survival rate of a less invasive stabilization system—distal femur (LISS) used to stabilize distal femoral fractures revealed that 23 out 107 fracture repairs required revisions and 4 of the revisions were caused by implant loosening (22). Another recent study in orthodontics evaluated 457 rough surface dental implants supporting 71 implant-supported fixed complete dental prostheses (52 patients) and found out that survival rates after 5.2 years was 98.7% (23). Another important finding in this study was a 10-year implant-based recession rate of 77% and a 10-year implant-based peri-implantatits rate of 20% (23).

## Osseointegration

Several biologic stages for osseointegration have been identified. These stages include the formation of a hematoma, development of mesenchymal tissue, the formation of intramembranous (woven) bone, and formation of lamellar bone (24–29). The first stage is initiated by bleeding caused by the insertion of the implant. Red blood cells, platelets, and inflammatory cells, such as polymorphonuclear granulocytes and monocytes, reach the surface of the implant by extravascular migration from the local bone vasculature and bone marrow cavity (7). These components of blood become entrapped on the implant's surface and their adhesion is followed by their activation which results in the secretion of cytokines and other growth factors, such as insulin-like growth factors (IGF I and II), FGF, TGF-β, and PDGF (7, 30–32). BMP 2 and 7, belonging to the TGF-β family, have gained a special interest in orthopedic research due to their excellent osteoinductive properties. Clotting is initiated by coagulation factors and extrinsic coagulation initiator tissue factor expressed by the osteoblasts (33). Activation of platelets induces several morphological and biochemical changes consisting of adhesion, aggregation, and induction of phosphotyrosine, an increase in intracellular calcium, and hydrolysis of phospholipids (7). Activated platelets and inflammatory cells secret chemoattractants which recruit fibroblasts, to ultimately shape a fibrin matrix that serves as a biologic and osteoconductive scaffold, which stimulates osteogenic cells to produce bone around the implant (i.e., osteoinduction) (7). Coagulation begins within 1 hour after implantation injury and granulation tissue starts being formed within 2 hours after the injury (34).

The fibrin matrix created on the surface of the implant is gradually transformed into a matrix that consists of poorly mineralized osteoid tissue structurally similar to bone cement lines and laminae. This matrix forms a continuous, ~0.5-mm-thick layer comprised mainly of calcium, phosphorus, osteopontin, and bone sialoprotein (25). This transformation process starts within 24 h after implantation and is primarily mediated by macrophages, which stimulate wound vascularization, migration of mesenchymal stem cells, and clearing the dead cells (35, 36). This thin layer of osteoid tissue on the implant's surface is gradually calcified by osteoblasts which additionally synthesize and secrete collagen (25). The collagenous matrix mainly contains type I collagen (28). Calcification is followed by the invasion of the osteoid tissue with the endothelial cells and mesenchymal stem cells in the non-calcified spaces (26). Vascularization of the osteoid, brought about by the invasion of endothelial cells, is a vital part of osteogenesis and impacts osseointegration substantially (27, 34). Osseous fragments (35–220 μm) created during the implantation process are also incorporated into the new regenerated interface (34).

Osteogenesis occurs directly on the surface of the implant (*direct osteogenesis*) and the margin of bone (*distance osteogenesis*). Both processes occur simultaneously and are separated by a clear line of demarcation (37). Mineralized osteoid tissue is gradually transformed into woven bone, which not only fills space but also maintains the integrity between the host bone and the implant, providing early mechanical support to the host bone during loading (27, 34). Woven bone also provides a scaffold for cellular attachment and deposition of bone (27, 38). These early processes related to the formation of bone begin as soon as 10 days after implantation (34). Woven bone begins to gradually remodel into compact, lamellar bone within three months after implantation (34). During this phase of remodeling, new osteons encircle the implant with their long axis parallel to the implant's surface. Osseointegration of an implant is complete when a thin layer of bone containing osteoclasts, osteoblasts, mesenchymal stem cells, lymphatic, and blood vessels surround the implant. This layer of bone can extend up to 1 mm from the surface of the implant (2, 38).

## Factors Positively Affecting Osseointegration

Osseointegration of an implant is a complicated and dynamic process positively influenced by numerous factors. Those factors can be distinguished into implant-related and host tissue-related components. The implant-related factors include the topography of the implant, geometric shape, length, diameter, material composition, interface distance, mechanical, and architectural properties, and implant micro/macro-motion (15, 28, 39–42). Implant osseointegration can be positively influenced by bioactive surface coatings such as hydroxyapatite or growth factors (43, 44). The host tissue-related factors include the quality of adjacent bone (45) as well as adjuvant treatments, such as bone grafting and application of an osteogenic coating (46–48). Several systemic pharmacological agents have been described to be of importance in osseointegration, among them, simvastin (HMG-CoA reductase inhibitor) and bisphosphonate (inhibitor of osteoclastic-mediated bone resorption) (49, 50).

One of the most important implant-related factors, which has a vital effect on the integration of the implant with adjacent bone, is the macroscopic and microscopic topography of an implant. A rough surface of the implant stimulates osseointegration through several mechanisms (39). The roughness of the surface activates the proliferation and differentiation of osteoblasts by activating integrin receptors that bind to the arginine-glycine-aspartate (ArgGly-ASP or RGD) sequences/domains of proteins (51, 52). Arg-Gly-Asp or RGD are expressed in several proteins found in the bone matrix, including collagen I, fibronectin, osteopontin, and bone sialoprotein. After implantation, proteins are bound to the surface of the implant and promote cellular adhesion through a ligand-receptor reaction (52, 53). Activated integrins regulate phosphokinase C (PKC) and A (PKA) through the phospholipase C and A2 pathways (54). Increased levels of PKA and PKC stimulate osteoblasts response to the systemic hormone—calcitriol (1,25-(OH)2D3), resulting in osteoblasts differentiation into osteocytes and secretion of differentiation factors, such as TGF-ß and PGE_2_ (51).

Rough surfaces stimulate greater expression of bone formation indicators, such as osteocalcin and alkaline phosphatase, as compared with smooth surfaces (48, 55). Roughness also increases the area of the implant in contact with host bone [bone-to-implant contact (BIC)], thereby improving primary stability (56, 57). Microscopic pores in an implant, with the size of 80 μm or greater (up to 250 μm), enhance the direct formation of bone on the implant's surface (58). Common methods described to increase the roughness of the implant's surface include coating the implant with hydroxyapatite or titanium beads, grit blasting, additive manufacturing (AM), plasma spraying (PS), physical vapor deposition (PVD), machining, laser treatment, anodic oxidation, sol-gel, chemical vapor deposition (CVD), acid etching, alkali treatment as well as cellular and protein coating improves roughness of the material (39, 56, 58).

Additive manufacturing, also known as three-dimensional printing has gained significant attention for application to orthopedic research as a method for filling complex bone defects. Using this method, the implants can be designed based on the complex anatomy of individual patients (59, 60). The scans are obtained by diagnostic imagining modalities, including magnetic resonance imaging (MRI), modern multi-row detector computer tomography (MDCT), computed tomography (CT), X-rays, or three-dimensional scanners. Obtained scans are imported into the computer system, where using sophisticated software, computer-aided designs (CAD) are created and printed with a three-dimensional printer. The scans obtained with diagnostic imaging allow to evaluate complex anatomy of the defects and create bone fillers which will fill them with high accuracy. Several bioprinting technologies have been described, including inkjet bioprinting, laser-assisted bioprinting, extrusion bioprinting, and stereolithography bioprinting (61–64). The specific design of bio-ink used for 3D printing is vital for implant integration. Bio-ink may or may not contain the cellular material, depending on the method of printing used. The average cell viability in bio-ink has been reported between 40 and 95% (60). The most important aspects of bioprinting include printing resolution, pressure applied on the bio-ink, time required for dispensing the bio-ink, viscosity of the bio-ink, post gelation strategy as well as crosslinking methods (60). The most widely used materials for 3D printing of bone include polycaprolactone (PCL), tricalcium phosphate, and hydroxyapatite (HA) (65–67).

Recent studies have shown that osseointegration is further enhanced when an implant has multiple types of surfaces, such as a combination of micro- and nano-topography. Nano pores positively influence the proliferation and differentiation and adhesion of osteoblasts (68). Implants with decreased thread pitch have improved mechanical stability and therefore result in enhanced osseointegration (69). Commonly used thread shapes are square, buttress, reverse buttress, V-shaped, and spiral-shaped (28). Square thread provides the greatest BIC and primary implant stability as well as experiences the lowest shear forces (70). Recently described trapezoidal thread design resulted in even faster osseointegration when compared to the conventional designs (71).

A small amount of micromotion between the implant and surrounding bone (30 μm) has been associated with enhanced osseointegration, however, motion >150 μm significantly impairs osseointegration (24, 72–75). The size of the gap between the implant and bone also influences osseointegration. Poor bone formation or even bone resorption has been observed, when an implant too tightly contacts (compresses) bone, whereas the presence of a small gap between the implant and host bone allows trabeculae to form, thereby supporting osseointegration. Gaps exceeding 500 μm result in the production of poor-quality bone and a delay in filling the gap (34, 35). Increased gap size substantially reduces BIC, thereby slowing osseointegration.

Material composition and its physical properties have a substantial influence on osseointegration. Ideally, the mechanical properties of the implant would be similar to that of the surrounding bone. Having a similar modulus of elasticity is most likely to ensure the preservation of the implant-tissue interface. Materials having a modulus markedly greater than that of human cortical bone (20 GPa) and trabecular bone (8 GPa), such as aluminum oxide (380 GPa), cobalt-chromium-molybdenum alloy (220–230 GPa), zirconia (210 GPa), and 316 L stainless steel (200 ± 20 GPa), are less likely to sustain long-term implant interface integrity as compared to materials, such as Ti−6Al−4 V titanium-aluminum-vanadium alloy (110 ± 10 GPa) and (PEEK) polyetheretherketone (3.6 GPa) or (CFR-PEEK) carbon-fiber-reinforced polyetheretherketone (18 GPa) which is more malleable (76–80).

Materials with markedly greater stiffness shield the surrounding bone from sustained loading. Bone remodeling follows Wolff's law that states that bone formation occurs in the loaded areas of bone whereas bone resorption occurs in the areas shielded from loading. The implants with greater modulus may shield bone from loading and may cause bone resorption resulting in reduced bone density and reduced cortical bone thickness (81–83). This phenomenon is known as stress shielding and it has a substantial negative effect on Osseointegration. Material modulus can be modified with the material composition, one of the examples includes distinct titanium alloys that result in a various amount of stress shielding (84). Recently designed titanium alloy (Ti−24Nb−2Sn) obtained a significantly lower modulus of 68 GPa (85). Although 316L stainless steel and titanium alloy have greater modulus than that of human cortical bone and trabecular bone, the more similar modulus to that of bone may contribute to improved long-term osseointegration of the implant (86, 87).

Properties of the material, which are expected to positively influence osseointegration, include high volumetric porosity (70–80%), optimal pore size, pore interconnectivity, pore geometry, high frictional characteristics, surface energy, and excellent biocompatibility of the material (88–90). The minimum pore size of 100 μm allows for mineralized bone formation and osteocyte migration within the implant (91). Pores with a size of 200–350 μm allow for the neovascularization within placed implants (92). Increased porosity and pore size have been found to reduce Young's modulus and yield strength of the materials (93). Appropriate pore interconnectivity allows for improved circulation of the interstitial fluid and nutrients within the implant (94). Pore geometry influences cell behavior and the cylindrical pores have been found to exhibit the best osteoconduction (95). A positively charged surface increases the hydrophilicity of the implant and therefore promotes protein adherence on implants surface as well as stimulates osteoblastic proliferation and differentiation (96). Porous tantalum (clinically pure tantalum) is the most osseointegrative metal currently used for constructing implants (88). Tantalum can be produced to mimic cancellous bone, has a low modulus of elasticity (3 MPa), high volumetric porosity (70–80%), high frictional characteristics, and the ability to form a self-passivating surface oxide layer (88, 97).

Dynamic research interest focuses on bioactive coatings (98). Bioactive coatings have been proven to improve material osseointegration. The most commonly used coatings containing calcium phosphates, including hydroxyapatite (HA), tricalcium phosphate (TCP), Whitlockite (WH), and octacalcium phosphate (OCP) (99–102). The osteoinductive properties of calcium phosphates are related to the process of layer degradation, during which free calcium and phosphate ions are released. This process results in increased local concentration, stimulating bone mineral formation on the surface of calcium phosphate (98). Furthermore, they enhance cell adhesion augmenting the adsorption of extracellular matrix proteins on their surface (103). Calcium phosphate molecules can be manufactured as macro-sized molecules or nano-sized molecules (104–106). Despite many advantages of these coatings, there are several disadvantages, such as inferior mechanical stability (98). These coating materials have been therefore used in combination with others. Further examples include bioglass (e.g., 45S5 bioglass) coatings and silica-based coatings (e.g., Dicalcium silicate, Tricalcium silicate) (107, 108). Bioglass has been shown to induce an apatite layer formation on its surface (107). Silica-based materials on the other hand own their bioactivity to the silicon (Si) (107, 108). Bioglass and silica-based coatings can incorporate calcium phosphate molecules (109).

## Factors Negatively Affecting Osseointegration

Factors having a negative impact on osseointegration can also be categorized as those that are implant-related and those that are host tissue-related variables. The implant-related factors include excessive micromotion (> 150 μm), high interfacial strain, inappropriate porosity of porous coatings, biological debris, and debris produced from wear, corrosion, and manufacture of the implant (24, 74, 75, 88, 110, 111). Host tissue-related factors include low quality of adjacent bone, often caused by systemic diseases (112–115), radiation therapy (116), and some medications such as cyclosporine A, methotrexate, cis-platinum, warfarin, low molecular weight heparins, and non-steroidal anti-inflammatory drugs (NSAID) (116–123).

Zirconia, aluminum oxide, and cobalt-chromium alloy present greater resistance to material wear and corrosion as compared with titanium and tantalum (124–128). These material properties could be modified with novel manufacturing strategies, such as using ultrafine-grained materials, thermal oxidation, or laser texturing of the titanium implants. The formation of a stable oxidative layer on the pure titanium has improved its biocompatibility and resistance to corrosion (129). PEEK can further hinder osseointegration because of its smooth surface, lack of antibacterial activity, and occasional detachment of coatings (130).

Bone cement often is used to fill the interface between orthopedic implants and bone during arthroplasty procedures to eliminate gaps and immobilize implants. Most commonly they are made from bioinert polymethylmethacrylate (PMMA) (131). PMMA has been associated with aseptic loosening as implant failure occurs at the PMMA and bone interface. Impaired osseointegration results in peri-implantitis, characterized by the formation of a fibrous interface surrounding the implant causing aseptic loosening of the implant (110, 132, 133). The inflammatory etiology of aseptic loosening has been associated with increased osteoclastic differentiation as well as macrophages and giant cell migration into the implant and tissue interface. This results in inflammatory-mediated osteolysis around the implant (134). There are several causes of this process, however, it has been commonly associated with the toxic MMA monomers, microfractures caused by high PMMA viscosity, increased intramedullary pressure caused by PMMA, and thermal damage due to the exothermic polymerization (56°C) (135–137). The infection rate in the surgery site with the implants fixed with PMMA has been reported between 14.8 (THA)–16.8% (TKA) (138, 139). The MMA monomers can also cause severe bone cement implantation syndrome (BCIS) defined with hypoxia, hypotension, cardiac arrhythmias, increased pulmonary vascular resistance, and cardiac arrest (140). Newer orthopedic cement is made from calcium phosphates (CPC). There are fewer drawbacks associated with CPC, including the different rates of bone regeneration and CPC degradation, limit of tissue ingrowth due to pore size, lack of mechanical strength, and inflammatory reaction caused by the degradation of synthetic polymers (141).

Other factors having a negative impact on osseointegration are osteoporosis, rheumatoid arthritis, advanced age, nutritional deficiency, smoking, and renal insufficiency (112–115). All these factors lead to failure of peri-implant osteogenesis, resulting in a decreased number and activity of osteogenic cells, increased osteoclastic activity, the imbalance between anabolic and catabolic local factors, and impaired vascularization of peri-implant tissue (43).

The gold standard for osseointegration evaluation is histology (142). The BIC can be directly evaluated and measured from the histology slides, as bone tissue in contact with the surface of the implant. The process also can be evaluated *in vivo* by using mechanical tests such as resonance frequency analysis (RFA) or percussion testing (Periotest) as well as *ex vivo* by using these and other tests, such as a measure of peak reverse torque (PRT) and tests measuring forces to pull out, pushout, torsion, and bending of the implant-tissue composite specimens.

## Stability of the Implant in Bone

The bond between bone and implant (e.g., orthopedic screw) relies on primary and secondary stability. Placement of the implant in the bone should result in immediately inadequate primary stability, but the degree of primary stability is influenced by several factors, such as surgical technique, design of the implant, the texture of the implant's surface, loading, micro-motion, and quality of surrounding bone (143–146). Primary stability also has been defined as “mechanical stability,” because it occurs immediately after implant placement and it is not affected by osseointegration (147, 148). Primary stability can be easily evaluated under *in-vivo* as well as *ex-vivo* conditions because it is associated only with mechanical properties. Implants with poor primary stability experience micro-motion exceeding 150 μm (1, 24, 74, 75), which leads to increased tensile and shear stresses, which in turn, lead to fibroplasia characterized by the formation of a fibrous interface surrounding the implant (110, 133). Fibroplasia may compound the displacement of the implant further decreasing the likelihood that the implant will achieve secondary stability (110, 132–134).

One of the factors strongly correlated with primary stability and related to surgical technique is insertional torque. Surgical drilling weakens the surrounding bone as a result of microfractures that occur during drilling; also drilling causes increases in temperature in the bone adjacent to the drill holes and within adjacent tissue (149). Bone cell death has been shown to occur when the tissue temperature of bone exceeds 47°C for 1 min or more (149). Surgeons commonly create undersized drill holes when preparing bone for placing an implant to create a radial preload compression force around the implant during placement. This practice increases insertion torque during placement of the implant and increases primary stability. In orthodontic implant surgery, research suggests that primary stability is optimized when the drill hole/implant disparity is no more than 10% smaller than the outside diameter of an implant (150). Decreasing the size of the drill hole to 25% of the outside diameter of the implant has not provided additional primary stability and would be expected to increase trauma to the surrounding bone during implantation (microfractures, excessive compression compromising vascular tissues) (150).

Placing a dental implant using high insertional torque has been found to result in microfractures and subsequent bone resorption; lower insertional torque is osteoprotective, resulting in better osseointegration (151–156). However, orthopedic research examining differences in primary stability provided by self-tapping and non-self-tapping screws, found that self-tapping screws, because they are placed using a significantly higher insertional torque than that required to place a non-self-tapping screw, provide greater primary stability than do non-self-tapping screws (11, 143, 157–159). The higher insertion torque required to insert a self-tapping screw is attributed to the torque required to cut the threads in bone with the cutting flute at the tip of the screw (11, 143, 157). Non-self-tapping screws lack this cutting flute, and their placement must be preceded by using a tapping device to cut the threads in the bone guide hole (11, 143, 157). The longer thread of the tapping device creates a micro gap between the screw and the bone, which results in a significantly lower insertion torque. This gap contributes to micromotion at the bone-implant interface resulting in increased interfacial strain (145, 146, 151, 157). This micro gap decreases the BIC, thereby decreasing the primary stability of the implant (160).

Orthotopic discrepancies between implant type and type of bone (appendicular, axial, craniomaxillofacial) result in different optimal insertional torque recommendations for placing these purpose and site-specific implants (e.g., dental implant, orthopedic screw). These variations can be explained largely by the differences in the bone into which the implants are placed and the design of the implants. The craniomaxillofacial bones are formed by intramembranous ossification, have characteristics of cancellous bone, and have less bone mineral density (BMD) as compared with long (appendicular) bones which are comprised primarily of compact cortical bone (161, 162). Bone-implant contact, insertion torque (IT), and primary stability of the implant and are strongly positively correlated (163, 164). Bones having lower BMD, such as craniofacial bones, typically have lower peak IT as opposed to load-bearing long bones which have much greater BMD. Bones with lower BMD are more prone to the destructive effects of high IT. A high IT use to insert an orthodontic implant may not result in optimal BIC and the primary stability of the implant may be compromised. Bones having greater BMD are more resistant to damage caused by high IT and are likely to achieve high BIC, thereby increasing the primary stability of the implant.

Primary stability can be evaluated under *in-vivo* and *ex-vivo* conditions by using common mechanical testing methods, such as resonance frequency analysis (RFA), percussion testing (Periotest M, Medizintechnik Gulden, Germany), IT, and cutting torque resistance analysis (CRA) (165–176). Measures of primary stability of an implant, including pullout, pushout, and bending, can be evaluated under *ex-vivo* conditions and in select *in vivo* applications (177–185).

**Figure 1:**
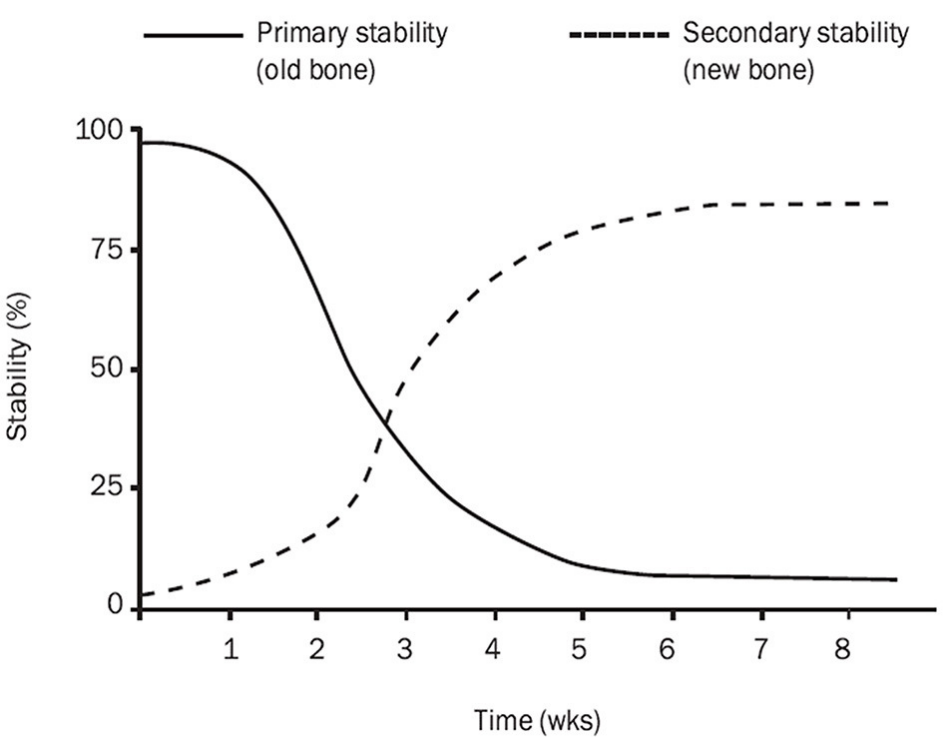
Implant stability in the bone. The graph presents gradual changes in primary (mechanical) and secondary (biological) stability of an implant placed in the bone. Primary and secondary stability influence on total implant stability. There is a decrease in total stability between 2 and 4 weeks after implantation. Graph imported from Raghavendra et al. (10).

Secondary stability increases as new bone is formed around and especially in contact with the implant (Figure 1) (10). Secondary stability is required for the long-term stability of the interface between the implant and bone and it is brought about by the process of osseointegration (147, 153). Secondary stability has been termed “biological stability” (147). The same factors impacting osseointegration equally affect secondary stability as both processes are interdependent and occur simultaneously (4, 23–29).

Studies have found a significant association between primary and secondary stability of an implant (147, 151, 153). A recent meta-analysis reported a significant, positive correlation (R^2^ = 0.847) between primary and secondary stability of dental implants as measured with RFA (153). Other studies looking at the long-term integrity of dental implants, with or without primary stability, found that primary stability is not the sole factor influencing osseointegration (146, 147). One study classified the primary stability of dental implants into four categories: including no rotation [1], light rotation with a feeling of resistance [2], rotation without resistance [3], and rotation combined with lateral oscillation [4] (186). Implants assigned to the first category were defined as the implants having adequate stability, whereas implants assigned to the second, third, and fourth category were defined as implants having no stability (186). An unstable dental implant has also been defined as an implant that rotates when a removal torque of <10 Ncm is applied and has slight lateral mobility (187). In a recent study examining the fate of 3,111 dental implants, survival rates after 3 years were 79.8% for those implants that had no primary stability (71 out of 89) and 93.4% (2823 out of 3022) of implants with primary stability (188). A more detailed analysis of the implants lacking primary stability revealed a significant difference in the incidence of survival between implants coated with hydroxyapatite (HA) (91.8% survival) and those not coated with HA (53.6% survival) (188), confirming the importance of texture of the implant's surface during the period of osseointegration. This finding shows that bioactive coatings such as HA may improve osseointegration, even for those implants lacking primary stability (189).

## Surgical Implant and Cartilage Interface

Implants placed within cartilage include tissue regenerative scaffolds placed within chondral or osteochondral defects. Those scaffolds aim at filling the defects and providing a surface on which the chondrocytes can attach, proliferate, and grow. The integration of implants within articular cartilage is challenging, because of its avascular nature, dense proteoglycans extracellular matrix (EM), and high compressive and shear forces applied to articular surfaces during physiologic motion of a joint (190, 191). The process of implanting tissue typically results in the death of chondrocytes adjacent to the implant (191). Also, cells capable of facilitating regeneration, such as recruitment of chondrogenic progenitor cells from circulating blood and marrow and resident chondrocytes, have limited ability to effect regeneration because these cells are unable to migrate through the dense EM. These factors negatively affect the process of integration.

Articular cartilage resurfacing with allogeneic or autologous implants is a viable approach to restoring physiologic function. In general, articular cartilage tissue is unable to fully regenerate defects when the lesion diameter is larger than 6 mm (192). Osteochondral lesions heal more readily than do cartilaginous lesions because of the exposure of subchondral bone which improved vascular and cellular migration (190). Hemorrhage from subchondral bone and subsequent formation of the blood clot formed after the osteochondral injury has been shown to fill defects up to 23 mm in diameter (190). The subchondral blood is known to provide growth factors, mesenchymal stem cells, and platelets necessary for regeneration. Defects in hyaline cartilage initially are repaired with fibrocartilaginous tissue (190). Fibrocartilage is mechanically and functionally inferior to hyaline cartilage largely because of the lower ratio of collagen I to collagen II and because it contains less aggrecans (193). A defect filled with fibrocartilage may appear to have healed, but the inferior nature of the fibrocartilage repair tissues results in deterioration of the tissue. Fibrocartilage is susceptible to microcracking which occurs around margins between the repair tissue and adjacent hyaline cartilage. This, ultimately, results in full-thickness fissures (194–196). Continuous transition at the interface of the implant and the cartilage into which it was placed is vital for the integration of the implant and retention of viable chondrocytes (190).

The integration of an implant begins with injury to the tissue in which it is implanted. In two *in-vitro* studies, implantation resulted in a zone of cellular death characterized by a band of necrosis 100–200 micrometers wide (197, 198). This band of necrosis was followed by gradual cellular apoptosis for 14 days, resulting in an extension of the band of necrosis of up to 400 micrometers from the center of injury (197, 198). Cells surviving outside the necrotic zone repopulate the matrix, but they only can attach to the same lacunae as daughter cells and cannot invade the remaining matrix (198). The limitations in the migration of resident chondrocytes suggest that inserting an implant containing viable stem cells or chondroblasts/cytes could enhance the process of integration (195, 199, 200). According to one study, autografts should contain between 5 and 30 million chondrocytes, depending on the size of the defect (200). The source of new chondrocytes is important (auricular cartilage, tracheal cartilage, etc.) as well as the source (autograft, allograft, xenograft) from which the chondrocytes are harvested (195, 199). The presence of even a small number of senescent cells within a cartilaginous graft has a negative influence on regeneration because these cells have pro-inflammatory properties, express a catabolic phenotype, and release metalloproteinase into the tissues (201). Cellular apoptosis can be inhibited with caspase inhibitors, such as ZVAD-fmk, but, to date, the use of caspase inhibitors to positively influence repair has not been rewarding (202).

The integration of an implant within cartilage is dependent on collagen fibers attaching to the surface of the implant and on migration and repopulation of the implant-cartilage interface with chondrocytes. An implant placed in subchondral bone achieves greater integration, and hence greater stability, than does one placed in cartilage alone (203). The size, modulus of elasticity, coefficient of friction, and Poisson's ratio of the implant are important for long-term chondrointegration (203). Inserting an implant with a diameter of no more than 5-mm minimally damages tissue and usually results in the successful incorporation of the implant into the surrounding tissue (203). Chondral and osteochondral implants that have a low material modulus and a high coefficient of friction cause more damage to the interface due to greater shear stresses related to joint motion as compared with implants with a high material modulus and a low coefficient of friction. An increase in Poisson's ratio is however associated with more damage to surrounding tissue when the implant is placed in an osteochondral defect as compared with implants placed in cartilage only defect. The differences in coefficient of friction and Poisson's ratio between the cartilage and placed implant have been explained with a different organization in the distribution of collagen fiber, as well as different collagen content and collagen crosslink density (203).

The collagen network at the implant-tissue interface is important (190). The process of integration involves the direct attachment of collagen fibers to the surface of the implant (190). The fusion of different types of cartilage, in different stages of development, may lead to collagen crosslinking catalyzed by lysyl-oxidase which insolubilizes ECM proteins and impairs the integration process (204). Modulating the crosslinking process before implantation, by using β-aminopropionitrile stimulates maturation of collagen and increases the adhesive strength of the collagen (205). Disruption of collagen fibers, either through injury or enzymatic digestion, accelerates chondrocyte proliferation and markedly increases their mobility resulting in implant and cartilage interface repopulation and enhancing integration (206, 207). The most common methods of enzymatic disruption of collagen fibers include treating the cartilage, into which the implant is placed, with collagenase, chondroitinase ABC, trypsin, or hyaluronidase (207–214). Enzymatic treatment also prolongs the synthesis of proteoglycans (211). Chemotactic agents, such as insulin-like growth factor-1 (IGF-1), and recombinant human bone morphogenetic protein−2 (BMP-2), boost cellular migration (214, 215).

Several adhesives have been shown to improve the stability of cartilage implants in tissue, the most commonly used being fibrin glue (216). Chondroitin sulfate biopolymers chemically modified with methacrylate and aldehyde groups have been used to connect biomaterials with tissue proteins (216). Collagen adhesin (CNA), a bacterial surface protein synthesized by *Staphylococcus aureus*, has been used by the bacteria to attach to monomeric chains of collagen (217, 218). CNA has a high binding affinity to collagen types I and II (217, 218). Other examples of biologic adhesives include cationized gelatin (219), RGD (220), combinations of various peptides, such as RGD, YIGSR, and IKVAV (221), and extracellular matrix proteins, such as collagen II and fibroblast growth factor (222). The molecules contained within the synovial fluid that provide lubrication, including PRG4 (SZP/lubricin/megakaryocyte-stimulating factor precursor) have also been positively associated with the integration of implants in cartilage (223).

## Testing Stability *in vivo*

Mechanical testing of implant stability conducted under *in-vivo* conditions includes resonance frequency analysis (RFA), percussion testing (Periotest M, Medizintechnik Gulden, Germany), insertion torque (IT), and cutting torque resistance analysis (CRA). These tests can be used to evaluate the micromotion of the implants or the resistance encountered during placement of the implant. The tests have been used, therefore, to evaluate primary as well as secondary stability.

The method used most commonly in orthodontic surgery to evaluate primary and secondary stability of an implant is resonance frequency analysis (RFA), developed and described by Meredith et al. (188). This method uses an L-shaped transducer connected to a vibrating element and receptor. The vibrating element applies a continuous and repeatable impact wave or a single impact force to the implant being assessed, and the receptor records the resonant frequency of the implant and surrounding bone (165, 166). Resonance was originally measured in Hz, but this form of measurement was replaced by a new measurement, the implant stability quotient (ISQ) measured with an Osstell™ device (Integration Diagnostics Ltd., Goteborgsvagen, Sweden) (167, 168). The ISQ of an implant is a graphical and numerical representation of implant stability. The ISQ ranges from 1 to 100, where an implant with ISQ of 1 is highly mobile, and one with an ISQ of 100 is maximally stable. An implant with an ISQ below 47 is considered to have insufficient primary stability (167, 168).

Another test used in orthodontics to measure primary stability is the Periotest^®^ (Periotest^®^, Siemens AG, Bensheim, Germany). The test was originally developed and described by Schulte to evaluate the mobility of native teeth (169). This device contains a metallic rod that applies controlled taps to the object being tested for stability. The object's response to tapping is measured by a sensor within a handpiece and it is converted to a Periotest^®^ value (PTV), which can range from −8 (low mobility) to +50 (high mobility) (13, 170). The PTV values of an osseointegrated implant should fall in the range of −5 to +5 (12). The PTV precisely reflects the BIC (171).

Cutting torque resistance analysis (CRA) was reported by Friberg et al. (172). This method used to determine primary stability measures the torque required to cut a thread into the drill hole at low-speed and does not evaluate secondary stability. This method gives valuable information about the quality of bone into which the implant is placed (172) but is used much less frequently to measure primary stability than is insertion torque (IT). The methods described above are used primarily in orthodontic research; research examining the accuracy of these methods to determine the primary stability of orthopedic implants is lacking.

Insertion torque (IT), a widely studied method of measuring primary stability in orthodontics and orthopedics, measures the torque at peak resistance during insertion of an implant (158, 166, 173) and, therefore, it does not assess the development of osseointegration or secondary stability of the implant (158). Recommendations regarding the optimal insertion torque for insertion of specific orthodontic and orthopedic implants to achieve optimum primary stability have been determined by using IT. Orthodontic studies, for instance, have determined the optimal torque for placing a dental implant to be 0.032 Nm (173). The IT values for inserting an orthopedic screw in a long bone are significantly higher and dependent on the size of the screw. For example, the optimal IT for inserting a 3.5-mm screw is 1.70 Nm, and that for inserting a 4.5-mm is 4.0 Nm for (174–176).

## Testing Stability *Ex vivo*

Secondary stability can be evaluated only after the implant had been placed in tissue. Osseointegration can begin as early as 10 days after implantation, and the process may take up to 3 months (21). The *in vivo* and *ex vivo* mechanical tests can be used to measure secondary stability and those used most frequently are described in the paragraph above. Testing not only the osseointegration of the implant but also the integrity of the entire construct requires *ex vivo* testing. *Ex vivo* testing of pullout, pushout, torsion, and bending requires the use of a mechanical testing system, such as the Instron machine (Instron, Norwood, MA). Peak reverse torque (PRT) can be also measured using a torque measuring screwdriver.

PRT measures the torque required to break the bond between bone and an osseointegrated implant and has been used successfully to test secondary stability of orthodontic and orthopedic implants (11, 12, 144–147, 170, 224). It is a very sensitive method used, indirectly, to evaluate the strength of the implant-tissue interface of the orthodontic as well as orthopedic implants (11). One study used this technique to evaluate the osseointegration of the self-tapping and non-self-tapping screws placed within a dynamic compression plate (DCP) used to stabilize segmental tibial defects for 60 days (11). The study found that self-tapping screws obtained greater peak reverse torque results consistent with greater osseointegration as well as that the non-uniform distribution of loading within the dynamic compression (DC) plate negatively affected the secondary stability of the screws placed proximally to the defect (11).

The following methods of mechanical testing methods discussed in this literature review are used to test entire constructs, such as an orthopedic plate and screws used to repair a long-bone fracture (205). Tests such as these must be conducted *ex vivo* because they aim to test the stability of a construct by stressing the construct until the construct fails (143, 177–179). Pullout tests have been commonly used to test the stability of orthopedic implants (143, 177–179). Tensile stress is applied to an implant or entire construct in a testing frame until the implant breaks or the implant is pulled from the tissue into which it has been implanted becomes displaced (177, 178, 180, 181). The rate of displacement must be adjusted according to the method of testing a specific construct.

The most important values examined by mechanical testing are maximum tension load, actuator displacement, and construct stiffness. The maximum tension load is the maximum tensile load measured at the point the implant or construct fails (181). The actuator displacement is the distance measured between the original position of the implant within the construct to the position of the implant at the end of mechanical testing, and construct stiffness is the calculated load over the slope of displacement (181).

Another test used to evaluate an implant alone or the entire construct in bone or soft tissue is torsional loading (177–180). The torsional load applied to an implant alone or the entire construct causes the implant or construct to rotate about an axis, creating a complex composition of internal stresses involving compression, tension, and shear (180). These stresses are greatest at the surface of the tested specimen and are reduced toward the specimen's neutral axis. The maximum shear stresses act on a plane perpendicular and parallel to the neutral axis, whereas the maximum normal tensile and compressive stresses act 45° to the neutral axis of the tested specimen. A recent study, using a human cadaveric model, comparing the stability of interference screws with that of unicortical buttons used to reattach the biceps tendon by testing torsional loading found the unicortical button provides higher stability of fixation (180).

*Ex vivo* testing of orthopedic implants has included bending tests (177, 182–185). Bending tests, in addition to generating bending stress, also generate compression and tension stresses (180, 185). Compression stress is applied to the upmost surface of the implant during bending, adjacent to the actuator, and tension is applied to the opposite side, far from the actuator (180, 185). Bending tests have been used to test the entire constructs, rather than the implant alone (182–184). Mechanical testing devices are capable of conducting two methods of bending including −3- and 4- point bending.

The difference between 3- and 4-point bending is related to the number of loading points attached to the actuator (184). When conducting a 3-point bending test, the actuator contains one loading point between two holding points, thereby creating three points of stress on the implant or construct being tested. The bending moment is concentrated in the area directly beneath the single loading point, resulting in highly focused bending stress (184). When conducting a 4-point bending test, the actuator contains two loading points and two holding points, thereby creating four stress points (182–184). The bending moment is equally distributed between two loading points, increasing the area of distribution of bending stress (184).

This literature review presents many several options for mechanical testing of the stability of osseous or soft tissue constructs or implants alone. Many tests can be used in a simple load to failure mode or in the cyclic relaxation-and-creep mode. In some situations, cyclic tests mimic *in vivo* conditions better than load-to-failure testing.

## Conclusions

Surgical implantation injures tissue adjacent to the surgical implant. Improved healing through enhanced cellular repair enhances the stabilization of the implant. Bleeding is the first step of healing of all injured tissues and it has a direct influence on the progression of healing. Poor vascularity of cartilage significantly hinders the integration of an implant. This literature review describes novel implants designed to be better integrated into cartilage and bone. Current knowledge reveals that the more closely the design of an implant mimics the native structure the more likely is the implant to become integrated. When the implant cannot closely mimic the native structure, coating it with a bioactive compound may stimulate the integration and successful outcome for the patient.

## Author Contributions

RG conducted the literature search and wrote the manuscript. JS, MD, DH, and P-YM supervised the literature search as well as reviewed the manuscript. DA supervised the literature search and manuscript writing as well as reviewed the manuscript. All authors have read and agreed to the version of the submitted manuscript.

## Conflict of Interest

The authors declare that the research was conducted in the absence of any commercial or financial relationships that could be construed as a potential conflict of interest.
